# The robustness of some Carboniferous fossil leaf venation networks to simulated damage

**DOI:** 10.1098/rsos.240086

**Published:** 2024-05-01

**Authors:** Luke Mander, Hywel T. P. Williams

**Affiliations:** ^1^ School of Environment, Earth and Ecosystem Sciences, The Open University, Milton Keynes MK7 6AA, UK; ^2^ Computer Science, University of Exeter, Exeter EX4 4QE, UK

**Keywords:** leaves, network evolution, percolation processes, herbivory, plant and insect co-evolution

## Abstract

Biological networks vary widely in their architecture and functional properties. Branching networks are good for transportation efficiency, while networks including loops offer good resistance to damage, and examples of these two topologies are found in leaf venation networks. The first plants with reticulate (loopy) leaf venation evolved in the Pennsylvanian of the Carboniferous, but the responses of different venation network architectures from this time period to damage are currently largely unknown. Here we address this issue with a computational analysis of venation network robustness that is focused on fossil leaves from the Pennsylvanian. We attacked fossil venation networks with simulated damage to individual vein segments and leaf blades. For both types of attack, branched venation networks are the least robust to damage, with greater robustness shown by the net-like reticulate networks found in the Pennsylvanian. A living angiosperm *Betula alba* was the most robust in our analysis. This may highlight a role for resistance to damage in the evolution of reticulate leaf venation in the Carboniferous, but further work is needed to answer the broader question of why reticulate leaf venation first evolved in the Pennsylvanian.

## 1. Introduction

**Figure 1.**
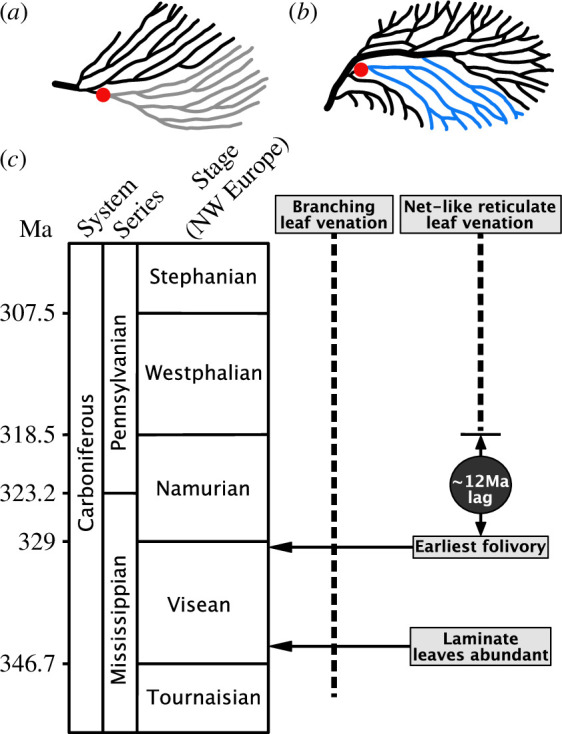
(*a*) In a branched network, in this case *Rhacopteris lindsaeformis*, an attack (red dot) can disconnect large portions of the venation network (grey veins). (*b*) In a net-like reticulate network, in this case, *Reticulopteris muensteri* small morphotype, the presence of loops means that an attack (red dot) may not disconnect the venation network (blue veins). (*c*) Leaf venation and folivory during the Carboniferous showing the stratigraphic ranges of venation types from data in Taylor *et al*. [1] and Boyce and Knoll [2], and markers for the abundant presence of laminate leaves in the fossil record and the first occurrence of folivory from Iannuzzi and Labandeira [3]. Age of the Mississipian/Pennsylvanian boundary from Cohen *et al*. [4], ages of NW European Stage boundaries from Richards [5]. The time lag between the earliest folivory and the appearance of reticulate leaf venation was calculated from the base of the Westphalian to the earliest Serpukovian ~330 Ma [5] .

Leaf venation is a classic example of a biological distribution network. Topologies fall into two broad classes: dichotomously branching trees (figure 1*a*) and loopy networks (figure 1*b*). Among living plants, examples of branching networks are found in ferns as well as the seed plant *Ginkgo*, while loopy networks are typical of flowering plants and some ferns [6–8]. The fossil record shows that reticulate (loopy) leaf venation evolved multiple times independently among unrelated seed plants during the latest Palaeozoic and Mesozoic [9,10]. Reticulate venation is thought to be a key anatomical innovation that provides several advantages over branched venation [8]. Reticulate venation can (but does not exclusively) increase the vein length per unit area of a leaf, which can enable the enhancement of physiological parameters that can be beneficial to a plant such as leaf hydraulic conductance, stomatal density and conductance and gas exchange per unit leaf area [8]. Modelling work has shown that it also enables optimal transport within the leaf under conditions of fluctuating flow or damage [11,12]. We focus here on how the physical process of damage could have affected the earliest reticulate leaf venation networks, which first appear in the fossil record in the Pennsylvanian of the Carboniferous [2,7,13] (figure 1*c*), and discuss why reticulate venation evolved in the Pennsylvanian.

Currently, there are two hypotheses that have been advanced to explain why reticulate leaf venation first evolved in this time period, both centred on the adaptive role of reticulate venation in the context of a changing global climate. Leaves with reticulate venation first occur in the fossil record during a period in the Carboniferous that was characterized by pronounced glacial–interglacial climate cycles, which resulted in the loss of wetland habitats and high turnover among plant groups [14]. Given the physiological advantages of reticulate versus branched venation in living plants, it has been suggested that reticulate venation may have been an adaptation to dry climatic conditions at this time and that if ‘this group of plants (the pteridosperms) was being placed under increased physiological stress due to water availability, any improvement in the plant’s water transport system, for example by increasing the area of interface between the veins and the mesophyll would be an advantage’ [13]. Similar arguments have centred on the idea that reticulate venation would be advantageous for water supply in a Pennsylvanian atmosphere that was depleted in CO_2_ [6], Kull 1999 in [7].

However, neither of these hypotheses considers whether damage may have played a role in Carboniferous leaf evolution. Experiments on living plants have shown that reticulate networks are more robust to damage than branching networks. For example, when subjected to embolism, the branching venation network of the fern *Adiantum* suffered catastrophic failure as distal portions of the network were disconnected by single embolism events, but the reticulate venation networks of *Quercus* and *Eucalyptus* showed a gradual decline in function, because the loops allowed some parts of the venation network to remain connected to the petiole (a stalk that attaches a leaf to the plant stem) as embolism proceeded [15]. These experimental observations are supported by computational analyses of leaf venation networks in flowering plants, which demonstrate that reticulate venation networks have greater redundancy and are more robust to damage than their minimum spanning trees, which have a branching architecture [16]. Similarly, as noted by Sack and Scoffoni [8], it is clear that in a branching network, severing veins at the base of the leaf kills the distal lamina of the leaf [17] (figure 1*a*), but in a reticulate network ‘if a grazer [for example] chews a hole the transportation of solutes could take place around the injury by a kind of capillary by-pass mechanism that is absent in free-veined blades’ [18] (figure 1*b*). Physical processes that could have affected Carboniferous leaves include embolism [15, 19], vein blockage by bacterial colonization (in the manner shown for Pierce’s disease bacterium in living grapevines [20]); or breakage due to wind [21]. Additionally, on the assumption that the fossil record faithfully records the relative timing of the origin of reticulate venation and folivory (the consumption of living leaf tissue as found in *Triphyllopteris austrina*; Late–Early Carboniferous of Australia; Iannuzzi and Labandeira [3]; figure 1*c*), then it seems plausible that folivorous insects could have inflicted damage on Carboniferous leaves with reticulate venation as well as their branched precursors.

Based on these observations alone, it is tempting to suggest that the first appearance of reticulate venation in the Pennsylvanian could be an outcome of selection for resistance to damage. However, the plants that first evolved reticulate venation (the pteridosperm order Medullosales [9]) are now extinct and therefore beyond the reach of direct experimentation. Also, the architecture of their leaf venation networks is not directly comparable to that of living flowering plants. In particular, their venation is net-like, lacking hierarchy and multiple vein orders that can allow for denser venation networks [22]. Consequently, the responses of different Carboniferous venation network architectures to damage are unclear, and this limits the understanding of how these leaves functioned in their physical environment as well as the degree to which Pennsylvanian reticulate venation networks are more robust to damage than branched venation networks.

To address these issues, we have investigated how a small number of extinct Carboniferous leaf venation networks with diverse network architectures respond to damage. We have taken a computational approach because, as noted above, direct experimentation on extinct plants is not possible. In the broadest sense, our study is concerned with why complex networks evolve in nature, and our overall motivation is to understand why reticulate leaf venation networks first evolved in the Pennsylvanian. The design of the study we present here (a small dataset and the investigation of a single point in time) limits the degree to which any conclusive answers to these goals can be drawn from our results. Nevertheless, as a first step, we ask the following specific questions: (1) What is the response of the fossil networks we study to damage sustained at the level of individual vein segments and to damage caused by simulated folivory? (2) Does the robustness of reticulate networks found in the leaves of Pennsylvanian pteridosperms exceed that of a typical contemporary branched network, and further, how does it compare to the robustness of the complex venation network in a modern angiosperm?

## 2. Material and methods

Images of fossil leaves were extracted from the published literature (table 1). We selected seven foliage taxa from the Pennsylvanian, two with branched venation and five with net venation, and focussed on this period because ‘reticulate venation appeared in the Westphalian [within the Pennsylvanian]’ [2]. We focussed on these seven taxa because records of their entire venation networks are preserved and because qualitatively they represent a variety of different Carboniferous venation types. Additionally, they are all thought to have botanical affinities to the pteridosperms, which we focussed on because it is thought that despite uncertainty surrounding the botanical affinities of many Carboniferous foliage taxa, ‘pteridosperms are the favoured plant hosts on which Paleozoic folivory was launched’ [3]. We selected two additional end-member taxa (representing primitive and derived venation network architectures) for our analyses: *Rhacopteris lindsaeformis*, also a pteridosperm but which evolved in the Mississippian and has branched venation, and *Betula alba*, a modern angiosperm which has hierarchical reticulate venation (table 1).

**Table 1.** Botanical and stratigraphic details of the nine taxa are analysed here. Botanical affinity of *Linopteris subbrongniartii* and *Lonchopteris bricei* from Boyce and Knoll [2], *Lonchopteridium laxereticulosum* tentatively assigned to the Medullosales on the basis of its morphological similarity to *Alethopteris* (see [1]), *Reticulopteris muensteri* assigned to the Medullosales from the trigonocarpales of Zodrow and Cleal [13] (see Taylor *et al*. [1], p. 1028 for accepted orders within the Pteridospermophyta), affinity of *Neuropteris jongsmani* and *N. heterophylla* from Taylor *et al*. [1], affinity of *Rhacopteris lindsaeformis* from Bateman *et al*. [23]. Age of *L. subbrongniartii*, *L. bricei*, *L. laxereticulosum*, *N. jongmansi* and *N. heterophylla* from Boyce and Knoll [2], age of *Reticulopteris muensteri* from Zodrow and Cleal [13], age of *Rhacopteris lindsaeformis* from Osborne *et al*. [24].

taxon	botanical affinity	age	venation type	image source
*Betula alba*	Angiosperm (Fagales)	extant	heirarchical	ClearedLeavesDB.org [25]
*Linopteris subbrongniartii*	Pteridosperm (Medullosales)	Pennsylvanian	net	Laveine [26]
*Lonchopteris bricei*	Pteridosperm (Medullosales)	Pennsylvanian	net	Crookall [27]
*Lonchopteridium laxereticulosum*	Pteridosperm (Medullosales)	Pennsylvanian	net	Boureau and Doubinger [28]
*Reticulopteris. muensteri small*	Pteridosperm (Medullosales)	Pennsylvanian	net	Zodrow and Cleal [13]
*Reticulopteris muensteri*	Pteridosperm (Medullosales)	Pennsylvanian	net	Zodrow and Cleal [13]
*Neuropteris jongmansi*	Pteridosperm (Medullosales)	Pennsylvanian	branched	Cleal and Thomas [29]
*Neuropteris heterophylla*	Pteridosperm (Medullosales)	Pennsylvanian	branched	Cleal and Thomas [29]
*Rhacopteris lindsaeformis*	Pteridosperm (Lyginopteridales)	Mississippian	branched	Galtier [30]

Leaf venation images were manually traced using a vector graphics software package (Affinity Designer; https://www.affinity.serif.com) and a digital graphics tablet (Wacom One; https://www.wacom.com). In this analysis, we have termed the midrib of pteridosperm leaves and the primary and secondary veins of *Betula alba* as the ‘structural vein’ and have termed all other vein segments as the ‘venation network’. For pteridosperm leaves, the structural vein was traced with a 4 pt line and the venation network was traced with a 0.8pt line. For *Betula alba*, the primary vein was traced with lines that tapered from 16 pt at the petiole to 12 pt, 8 pt and then 4 pt at the leaf tip, the secondary veins were traced with a 4 pt line, and the venation network was traced with a 0.8 pt line. These line widths were selected as the closest qualitative match to the width of the vein segments in the source images. Veins were traced in segments, and a line segment was accepted if any part of it overlapped the vein in the source image, and was rejected and redrawn if any part of it fell outside the vein in the source image. Tracings of the structural vein and the venation network of each leaf were rasterized and stored as separate binary GIF files (electronic supplementary material, dataset S1), and rescaled to a standard magnification, with one pixel representing 10 µm, and vein segments in each venation network measuring three pixels in width.

**Figure 2.**
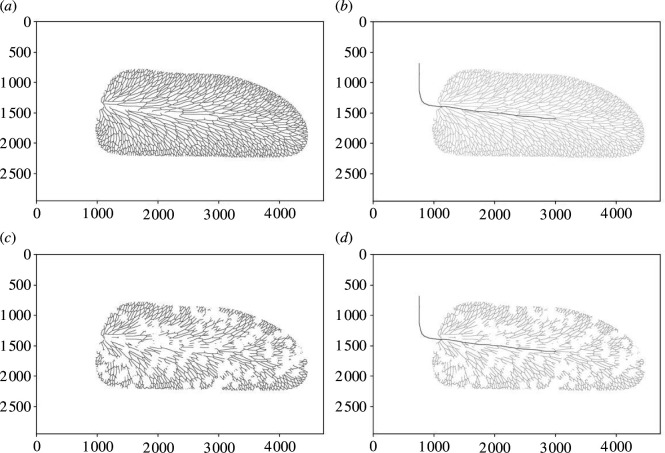
(*a*) Pristine venation network, in this case *Linopteris subbrongniartii*. (*b*) The venation network attached to the structural vein following an attack by random pixel removal from the venation network (in this example, 0.75 of the venation network is present and 0.03 of the venation network is connected to the structural vein following the attack). (*c*) A leaf blade attacked by removing circular holes from the leaf blade (in this example, 0.69 of the venation network is present following the attack). (*d*) Venation network attached to the structural vein following the attack (in this example, 0.46 of the venation network is connected to the structural vein following the attack). Each image in (*a*–*d*) is displayed in pixel units and each pixel represents 10 µm.

In general terms, our analyses of venation network robustness involve removing portions of a venation network and using connected component labelling [31] to measure the amount of the venation network that remains connected to the structural vein. This metric is a simple proxy for retention of a functional vascular transport system within the leaf. Specifically, our analyses begin with a pristine venation network (figure 2*a*). To identify the venation network within each leaf image, we labelled the connected components in each image by connecting pixels using an eight-connected neighbouring relation (cf. ‘blob extraction’ [31]). We protected the structural vein of the leaf and allowed damage only to the venation network, which we attacked in two different ways. Firstly, we attacked veins by randomly removing pixels from the venation network, then attached the structural vein of the leaf and counted the pixels in the connected region that remained attached to it (figure 2*b*). Removal of a single pixel from a single unbroken vein segment would not break the vein segment into two because of the eight-connected neighbouring relation. Disconnection of such a vein segment into two or more separate segments would occur as pixels are progressively removed. This attack is intended to represent some form of damage at the level of the vein itself, as might be produced by embolism [15, 19] or vein blockage by bacterial colonization [20], but we do not simulate any processes directly. For example, visual mapping of embolism spread through a leaf shows that initial embolism nucleation occurs in the largest diameter veins (the structural vein of this study) before spreading through the remainder of the venation network [15], but in our vein-level attack pixels are removed randomly from the venation network alone. Secondly, we attacked leaf blades by making circular holes 60 pixels in area in the leaf blade, which removes vein segments from the venation network (figure 2*c*). We then attached the structural vein of the leaf and counted the pixels in the connected region that remained attached to it (figure 2*d*). This attack is intended to simulate an idealized form of folivory, in which portions of a leaf blade and venation network are consumed by a herbivore. However, beyond hole feeding, we do not simulate specific types of leaf damage that result from other functional feeding groups such as margin feeding, surface feeding, skeletonization, piercing and sucking, galling and leaf mining [32–34].

The two attacks we have simulated represent generalized and contrasting types of network damage, and we do not attempt to directly simulate specific processes that are responsible for damage to venation networks. Consequently, our results likely do not perfectly match the tolerance of the taxa analysed here to damage sustained in the real world, especially given the range of environmental conditions (light regime, water availability and temperature) that a leaf is exposed to in the course of a growing season [35]. For example, in order to specifically measure how water transport capacity is affected by damage to the venation network, a model of fluid flow through the leaf would be needed. Such a model could be based either on Ohm’s Law and the analogy between the flow of current through an electrical circuit and the flow of water through a conduit [36] or on Hagen–Poiseuille calculations of flow rates in hollow tubes [37,38] (see discussion in [39]), in conjunction with the results of anatomical work reconstructing the hydraulic architecture of fossil leaves (for such work with living plants see Carvalho *et al*. [40] on *Ginkgo biloba* and Carvalho *et al*. [41] on *Populus tremula* x *alba*). Given the complexity of connectivity among xylem vessels, a model based on Ohm’s law may only suffice for an undamaged leaf and would require appropriate scaling of resistances based on vessel properties. A more appropriate approach may be that of Mrad *et al*. [42] who studied xylem hydraulics in *Acer* wood, and modelled flow through vessel lumens using the Hagen–Poiseuille equation and flow through intervessel connections by a superposition of Sampson and Hagen–Poiseuille flow resistances. Instead, our simulations are designed to explore the potential nature and range of damage responses in the taxa we have analysed, and also to inform the design of future computational [16] and experimental work [43] in this area.

For each taxon, we repeated each attack 250 times, randomly varying the proportion of pixels removed between 0 and 1 (for vein attacks) and randomly varying the number of holes between 0 and 2000 (for leaf blade attacks) to generate a distribution of attack outcomes for each taxon. Our key output metric is the proportion of the venation network that remains after the attack relative to the proportion of the network that remains attached to the structural vein of the leaf by some continuous path in the venation network. This approach is similar to analyses of network robustness based on percolation that show how the size of the largest component of a network changes as elements of the network are removed [44]. These computations were undertaken using a script written in the Python programming language (electronic supplementary material, dataset S1). We binned datapoints into 20 equal bins between 0 and 1, calculated the mean and standard deviation of each bin, and connected the mean values with a trendline. To give a single metric to quantify the overall robustness of each venation network, we calculated the sum of the 20 mean values to approximate the integral of the trendline. These computations were undertaken using a script written in the Python programming language (electronic supplementary material, dataset S1).

## 3. Results

**Figure 3.**
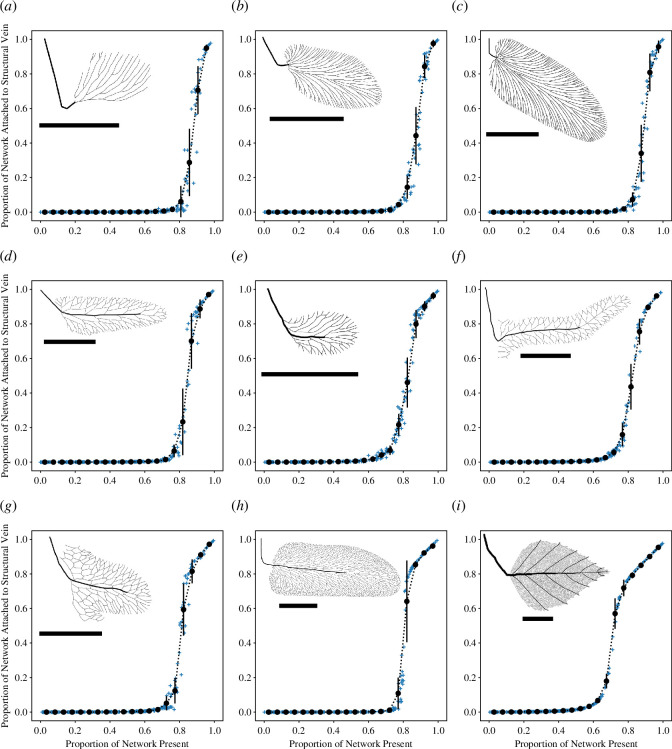
Response of venation networks to simulated vein-level attacks. Each plot shows the relationship between the proportion of the venation network that remains after the attack relative to the proportion of the network that remains attached to the structural vein of the leaf by some continuous path in the venation network. Crosses show the outcome of each individual attack, closed circles show the mean value of each bin, whiskers show the standard deviation of each bin, and a dashed line connects the mean values. The venation networks of leaves (*a–c*) are classified as branches, (*d–h*) as nets, and (*i*) is a hierarchical network. The nine taxa are (*a*) *Rhacopteris lindsaeformis*, (*b*) *Neuropteris heterophylla*, (*c*) *Neuropteris jongmansi*, (*d*) *Reticulopteris muensteri*, (*e*) *Reticulopteris muensteri* small morphotype, (*f*) *Lonchopteridium laxereticulosum*, (*g*) *Lonchopteris bricei*, (*h*) *Linopteris subbrongniartii* and (*i*) *Betula alba*. The thumbnails of each leaf are shown at different magnifications with a 10 mm scale bar.

**Figure 4.**
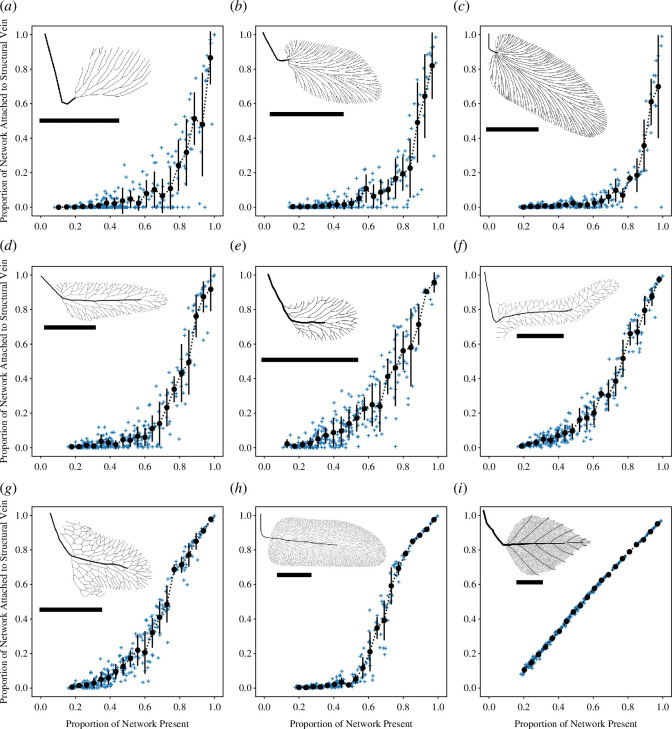
Response of venation networks to simulated leaf blade attacks. Details as in figure 3.

The results of our computations are shown in figures 3 and 4 (see electronic supplementary material, dataset S2 for the data used to produce these plots). The low-complexity end-member *Rhacopteris lindsaeformis* with simple branched venation is shown in figure 3*a*, while the high-complexity end-member *Betula alba* with hierarchical reticulate venation is shown in figure 3*i*. Between these two are the Pennsylvanian foliage taxa that have venation networks ranging in complexity and that include both branching and net-like architectures (figure 3*b*–*h*). Under a vein attack, all the venation networks analysed here show a collapse transition in which the function retained by the network rapidly decreases as the amount of damage sustained by the network increases (figure 3). For *Rhacopteris lindsaeformis*, the transition begins when ~0.95 of the network remains after damage, and the retained function drops to ~0.05 when ~0.80 of the network remains after damage (figure 3*a*). In contrast, for *Betula alba,* the transition begins when ~0.75 of the venation network remains after damage, and the retained function drops to ~0.05 when ~0.60 of the network remains after damage (figure 3*i*). The Pennsylvanian taxa that have branched venation networks show a rapid disintegration of the venation network similar to that of *Rhacopteris lindsaeformis* (figure 3*b*,*c*), while for the Pennsylvanian taxa that have loopy venation networks the collapse transition begins when ~0.85 of their venation networks remain after damage (figure 3*d*–*h*). There are some differences in the transition from a connected and functional network to a disintegrated and non-functional network among those taxa with loopy venation. The phase transition for *Reticulopteris muensteri*, for example, begins at ~0.85 (figure 3*d*), whereas for *Linopteris subbrongniartii* it begins at ~0.8, indicating that more of the venation network has to be removed in order to induce network collapse (figure 3*h*).

Under a leaf blade attack, the branched networks are vulnerable to catastrophic failure when very small amounts of the venation network are removed by a ‘lucky strike’ that disconnects a large proportion of the network (see also figure 1*a*), and for *R. lindsaeformis*, *N. heterophylla* and *N. jongmansi*, there is no evidence of a coherent and systematic collapse transition (figure 4*a*–*c*). The loopy venation networks of *R. muensteri, L. laxereticulosum*, *L. bricei* and *L. subbrongniartii* do not appear to be vulnerable to catastrophic failure as a result of ‘lucky strikes’ (figure 4*d*–*h*). Neither *Reticulopteris muensteri* nor *L. laxereticulosum* is characterized by a clear collapse transition (figure 4*d*–*f*), but the venation networks of *L. bricei* and *L. subbrongniartii* may show critical behaviour, with increased variability when ~0.80–0.60 of their venation networks remain after damage (figure 4*g,h*). The modern angiosperm *B. alba* shows near optimal (‘graceful’) network degradation, where the loss of network function is almost directly proportional to the damage intensity (figure 4*i*).

**Figure 5.**
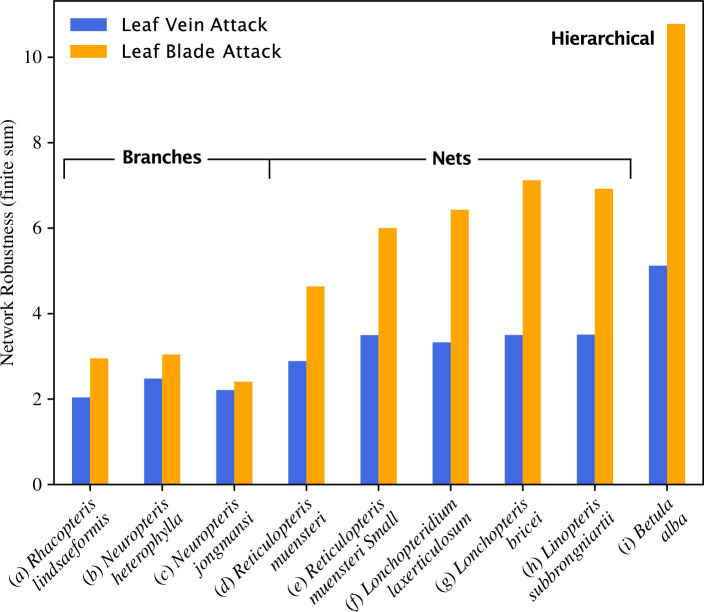
The robustness of each venation network analysed here to vein-level attack and leaf blade attack. (*a*) *Rhacopteris lindsaeformis*, (*b*) *Neuropteris heterophylla*, (*c*) *Neuropteris jongmansi*, (*d*) *Reticulopteris muensteri*, (*e*) *Reticulopteris muensteri* small morphotype, (*f*) *Lonchopteridium laxereticulosum*, (*g*) *Lonchopteris bricei*, (*h*) *Linopteris subbrongniartii* and (*i*) *Betula alba*.

The robustness of each venation network analysed here is summarized and compared in figure 5. The venation networks of the two end-member taxa in our analysis, *R. lindsaeformis* and *B. alba*, have the lowest and highest robustness, respectively, under a vein attack and a leaf blade attack (figure 5). For all taxa, venation network robustness was greater under a leaf blade attack compared to a vein attack, and this was most pronounced for *L. bricei* and *B. alba*. Taken together, branched venation networks were the least robust in our analysis, net-like architectures were more robust than branched networks, while the hierarchical reticulate network of *B. alba* was the most robust (figure 5).

## 4. Discussion

### 4.1. The connectivity of fossil venation networks

The fossil record of morphology is an archive of evolved design solutions to the problems faced by organisms living on Earth. Some of these solutions evolved during periods in Earth history characterized by conditions that are not known on Earth today, such as an atmosphere with a radically different composition, or are known only from organisms that are now extinct. In such situations, direct experimentation is not possible and this hampers knowledge of how certain morphologies functioned, which in turn, presents a barrier to understanding why certain morphologies evolved at certain periods in Earth’s history. In this article, we have attempted to generate understanding of how fossil leaf venation networks may have responded to damage by simulating network attacks computationally (figure 2, electronic supplementary material, dataset S1). We have investigated a small number of fossil leaves from the Pennsylvanian to examine how network robustness changed during the evolutionary transition from branching to reticulate leaf venation in the Pennsylvanian (table 1, figure 1*c*), and to compare the relatively simple venation networks of these extinct taxa to the highly complex venation network of a living angiosperm *B. alba* (figures 3–5).

In most analyses of this nature, some assumptions are made about morphological details for which there is insufficient data currently available, and in this study, we have made assumptions concerning the connectivity of overlapping veins in *R. muensteri* (e.g. figure 3*d*). In particular, we have interpreted all instances where veins come into contact with one another as fully functional reticulation—they represent loops in the leaf venation network in our analyses—but this has not been tested by cross-sections of veins to examine fine bundle anatomy in fossil specimens, and it is possible that some instances of contact between individual vein segments do not represent functional physiological loops that are required for effective fluid rerouting [13]. A similar situation exists with the branched networks of living ferns: ‘practically all ferns will be found to have occasional anastomoses of veins’ [18, p. 90], and with the branched networks of *Ginkgo biloba* where ~10% of leaves in a collection of 1065 possessed one or more instances of overlap between veins [45] and in which ‘the degree of union between anastomosing veins fluctuates widely’ [45, p. 408]. In the case of *Reticulopteris*, detailed anatomical work on well-preserved fossil leaves could clarify the situation, and a combination of anatomical and experimental work involving visualizing fluid flow with fluorescein could be employed to investigate isolated reticulation in ferns and *Ginkgo biloba* (see [12] for an example of such experimentation on *Ginkgo biloba* and *Citrus limon*).

### 4.2. The robustness of leaf venation networks to simulated damage

In general, our results are congruent with intuition (e.g. figure 1*a*,*b*), theory [11,12,16] and experimentation [15] that shows loopy network architectures are more robust to damage than branched architectures, which are vulnerable to catastrophic failure (figures 3–5). Despite this vulnerability, however, branched networks do not always completely collapse under folivory, and there is a degree of robustness under this form of attack (figure 4*b*). In particular, sections of the leaf removed around the margins by folivory will not cause catastrophic failure because only the distal portion of the lamina is removed. Indeed, the first occurrence of folivory in the fossil record (on *Triphyllopteris austrina*) consists of cuspate margin-feeding activity rather than isolated holes removed from the central portions of the leaf blade [3]. Nevertheless, even this type of folivory has the potential to cause large-scale disconnection of branched networks, and figures 3*d* and figure 4*d*,*f* in Iannuzzi and Labandeira [3] each demonstrate that marginal feeding has disconnected a substantial distal portion of branching venation network. All the fossil venation networks with net-like architectures analysed here, together with *B. alba*, appear resistant to catastrophic failure under simulated folivory (figure 4*d*–*i*). This likely reflects the effects of loops in the network, which provide redundancy and alternative pathways to the structural vein (figure 1*b*) [16]. Robustness may also be helped by the presence of a midrib in the Pennsylvanian reticulate leaf fossils as well as in the primary and secondary veins in the angiosperm *Betula alba*. These veins increase the number of contact points with the venation network, increasing redundancy and removing the single point of failure inherent in the architecture of a branched network. *B. alba* also has considerably higher vein density than the other taxa analysed here (as is typical for angiosperms), and although we do not quantify vein density as part of this work, it has been shown previously that ‘high-density reticulate minor venation provides damage tolerance’ [46, p. 1569].

### 4.3. Why the Pennsylvanian?

To what extent do our results inform understanding of why reticulate leaf venation first appears in the Pennsylvanian? Our results demonstrate that the branched and reticulate venation networks of the fossil leaves we have analysed here differ in terms of their robustness, both at the level of a vein attack and a leaf-blade attack (figure 5). While this highlights that resistance to damage could plausibly have played a role in the evolution of reticulate leaf venation, in order to conclusively answer this overall question, time series analysis of network robustness over a longer time interval with a more complete sampling of Carboniferous leaves is needed. It would also be helpful to consider other co-variates within such a time series. These could include vein density, which relates to the physiological performance of a leaf, and such an analysis could help to test whether increased robustness (this study) or greater physiological performance (either in a dry Pennsylvanian climate [13] or a CO_2_ depleted atmosphere [6], Kull 1999 in [7]) have greater explanatory power. Additionally, consideration of how network damage impacts physiological processes such as leaf gas exchange (as has been investigated with experimental herbivory in living desert plants by [43] who found no positive or negative influence of reticulation on the resilience of post-damage leaf gas exchange) could be facilitated by a model of fluid flow through Carboniferous fossil leaves, and would enhance understanding of how plant function changes as a consequence of network damage. Further work should also confront the possibility that neutral developmental diversity (presumably as in *Ginkgo biloba* [45]) could have produced the first reticulate leaf, and the neutral evolution of leaf developmental mechanisms [2,9] could also have resulted in a leaf with reticulate venation in the Pennsylvanian.

Finally, regardless of mechanism, following its origin in the Pennsylvanian, plants with reticulate leaf venation are relatively minor components of Carboniferous fossil floras [2], and in the specific case of Medullosan seed-ferns, the group of plants in which reticulate leaf venation first evolved, plants with reticulate leaf venation are not characterized by rapid diversification and are neither speciose nor ecologically dominant (see [1]). The innovation of reticulate leaf venation therefore did not translate into immediate ecological dominance, and it was not until the evolution of angiosperms in the later Mesozoic era that leaves with reticulate venation became abundant in extant and extinct vegetation. However, this group of plants also harnessed a suite of other key physiological and reproductive innovations that enabled their rise to ecological dominance [8,47,48] as part of the Angiosperm Terrestrial Revolution [49]. For extinct plants, the ecological value of the functions of reticulate venation—which includes increased robustness to damage, as demonstrated here (figure 5)—is therefore somewhat unclear, and perhaps reticulate venation networks in Palaeozoic leaves should be viewed simply as a viable alternative design solution to branched venation networks.

## 5. Concluding remarks

— When attacked at the level of individual veins, all the venation networks analysed here show critical behaviour whereby network function (measured by the proportion of the network that remains attached to the structural vein) rapidly decreases with an increase in the level of damage (measured as the proportion of the network that is retained following attack; figure 3). Branched venation networks are the least robust to a vein-specific attack, with greater robustness shown by the net-like reticulate networks found in the Pennsylvanian, while a living angiosperm *B. alba* was the most robust in our analysis (figure 5).
— In contrast to a vein-level attack, an attack on the leaf blade by simulated folivory does not result in a coherent and well-defined phase transition for branched venation architectures, and instead, there is a broader distribution of outcomes (figure 4*a*–*c*). These branched networks are vulnerable to catastrophic failure when an attack removes a key vein, but the net-like reticulate networks found in the Pennsylvanian are resistant to this type of collapse because they contain redundant alternative paths (figure 4*d*–*h*). Two of the net-like venation architectures we have studied may show critical behaviour under a leaf-blade attack (figure 4*g,h*) while the living angiosperm *Betula alba* shows near-optimal network degradation (figure 4*i*). In common to a vein-specific attack, branched venation networks are the least robust to a leaf-blade attack, with greater robustness shown by the net-like reticulate networks found in the Pennsylvanian, and the greatest robustness displayed by *B. alba* (figure 5).
— The branched and reticulate venation networks of the fossil leaves we have analysed here differ in terms of their robustness, both at the level of a vein attack and a leaf-blade attack (figure 5). This may highlight a role for resistance to damage in the evolution of reticulate leaf venation in the Carboniferous. However, the dataset on which our results are based is small in size (nine leaves in total) and our investigation considers only a single point in time. Additionally, our study lacks a model of fluid flow through the leaf that would be needed to measure how water transport capacity is affected by damage to the venation network. These elements of our study design limit the degree to which our conclusions can be generalized, and further work based on a more complete sampling of Carboniferous leaves and employing time series analysis of network robustness over a longer time interval is needed to answer the overall question of why reticulate leaf venation evolved in the Pennsylvanian.

## Data Availability

All data and code is available in the supplementary material [50].
